# Cryosurgery: Its Role in Liver Tumours

**DOI:** 10.1155/1993/18325

**Published:** 1993

**Authors:** K. E. F. Hobbs

**Affiliations:** University Department of Surgery The Royal Free Hospital School of Medicine Pond Street London NW3 2QG UK


*HPB Surgery*, 1993, Vol. 7, pp. 89–97
Reprints available directly from the publisher
Photocopying permitted by license only

© 1993 Harwood Academic Publishers GmbH
Printed in the United States of America

### HPB INTERNATIONAL

**EDITORIAL & ABSTRACTING SERVICE**

JOHN TERBLANCHE, EDITOR
Department of Surgery,
Medical School · Observatory 7925
Cape Town · South Africa
Telephone: (021) 47-1250 Ext. 2
Telefax (021) 448-6461

### CRYOSURGERY: ITS ROLE IN LIVER TUMOURS

### ABSTRACT

*Ravikumar, T.S., Kane, R., Cady, B., Jenkins, R., Clouse, M. and Steele, G., Jr. (1991) A 5-year study of cryosurgery in the treatment of liver tumours. Archives of Surgery;* **126**: *1520–1524.*

**●This report summarizes our 5-year experience with cryosurgery for *in situ* ablation of liver tumors. The liver was exposed with laparotomy, and the tumors were subjected to two freeze-thaw cycles using liquid nitrogen delivered by insulated probes; cryoablation was monitored with intra-operative ultrasonography. Tumor markers and computed tomography evaluated tumor response during long-term follow-up. From 1985 to 1990, 32 patients (19 men and 13 women) were entered into this study. The histologic characteristics of the tumors were as follows: colorectal, 24 patients; hepatoma, three patients; neuroendocrine, two patients; and others, three patients. After a follow-up period of 5 to 60 months (median follow-up, 24 months), nine patients (28%) remained disease free, 11 patients (34%) were alive with disease, and 12 patients (38%) died. The patterns of failure included liver and extrahepatic disease in 54% of cases, liver disease only in 32% of cases, and extrahepatic disease only in 14% of cases. In patients with "liver only" failure, recurrence at the treatment site occurred in three patients (9%). This study establishes the long-term effectiveness of cryosurgery in the treatment of primary and metastatic liver tumors. (Arch. Surg. 1991; 126: 1520–1524)**

### PAPER DISCUSSION

KEY WORDS: Liver secondary carcinoma, cryosurgery

This paper reports the use of cryosurgery in the treatment of liver tumours and raises two interesting issues: the value of cryodestruction as a technique for destroying malignant tumours in general and more specifically for the management of liver tumours.

Cryosurgery has been used successfully for many years for tumour destruction in surgical fields. Perhaps it has been used most successfully and enthusiastically for the treatment of skin cancers. Basal cell carcinomas and some epitheliomas can be treated with a reported cure rate of 97.6%. These results are comparable to surgical excision and radiotherapy[1,2]. There are two main advantages of cryodestruction over other treatments for skin lesions: frequently only one outpatient attendance is needed and healing is almost perfect, except around the eyes, with minimal scarring. Sometimes the only indication that any treatment has occurred is a residual area of depigmentation; this is often more marked in dark skinned patients.

Some studies report the value of the technique as a palliative symptomatic method of dealing with large inoperable tumours such as ulcerating carcinomas of the rectum. It has been shown to be of value in palliating many of the unpleasant symptoms of these lesions in patients for whom surgical resection was not possible[3].

Although its value has been investigated as a method for managing prostatic tumours, it fell into disrepute due to complications, especially urethrorectal fistulas. However in a recent report to the Society of Cryobiology, Dr Jeffrey Cohen and co-workers from Allegheny General Hospital, Pittsburgh point out that by refining the technique they observe few local complications and their early data suggests it is effective in destroying malignant tissue[4].

At the same meeting Dr Marcove and colleagues from Memorial Sloan Kettering Cancer Center report some favourable results for the technique in the management of bone malignancies[5].

Cryosurgery is a simple technique and there are several liquid nitrogen cooled cryosurgical machines on the market. There has been some engineering development of the probes recently and some are now fine enough and sufficiently insulated to be inserted into tumours. Most evidence suggests that two freeze-thaw cycles using a liquid nitrogen cooled probe will provide total destruction of any cells. Since it is so straight forward a procedure it is surprising that it does not attract more attention from clinicians.

Unfortunately most enthusiasts who advocate using the technique fail to carry out randomised studies designed to compare prospectively the results of cryosurgery and more conventional treatments of the same tumour.

This is the case in the management of liver tumours and this paper by Ravikumar and colleagues is another example. It set out to study the value of cryosurgery for treating patients with a mixed bag of primary and secondary liver tumours (mostly secondary) not suitable for surgical resection. The authors describe clearly how they use ultrasound guidance to watch in real time the growth of the ice ball through the malignant lesion. They and others[6,7] have shown previously that this is a very practical and effective technique for ensuring all the tumour is frozen. In this paper they show that they are able to totally destroy some malignant lesions in the liver by local freezing. However although the overall results of treatment in this group of patients, who were not amenable to surgery, were encouraging, there were no controls against which the effect of the cryosurgical treatment could be judged. By very nature of the fact that the patients treated were "inoperable" the tumours must have been quite advanced.

The subsequent discussion of this paper by others at the meeting to which it was presented centred on the treatment of these advanced and inoperable liver tumours. However in this situation the success or failure of local treatment is often determined by progression of untreated metastatic disease at other sites. Dr Gage says, "Findings of some reports have suggested that the survival of patients with metastatic carcinoma is extended by cryosurgical treatment. Whether or not this is true will require additional study"[2].

Certainly it seems that cryodestruction is a valuable therapeutic modality for treating liver cancers. It is safe and in one study of 60 patients with primary liver cancer treated by cryosurgery there were no complications of bile leakage, bleeding, infection or operative death[8].

I believe the next step now is to design a prospective study of the treatment of not inoperable lesions but small and operable tumours. It should compare surgical resection, cryotherapy and the other technique reported to destroy liver tumours, absolute alcohol injection[9]. To make such a study really meaningful it would be necessary, too, to include an untreated limb. However the ethics of this would need careful discussion. As a result of such a study we would really gain some idea of the value of cryodestruction in relation to the natural history of the disease and in comparison with other treatment modalities.

Professor K. E. F. Hobbs
University Department of Surgery
The Royal Free Hospital School of Medicine
Pond Street
London NW3 2QG
United Kingdom

### REFERENCES

1. Zacarian, S.A. (1983) Cryosurgery of cutaneous carcinomas. An 18 year study of 3022 patients with 4228 carcinomas. *J. Am. Acad. Dermatol.*, **9**, 947–956
2. Gage, A. (1992) Cryosurgery in the treatment of cancer. *Surgery, Gynecology & Obstetrics*, **174**, 73–92
3. Osborne, D.R., Higgins, A.F. and Hobbs, K.E.F. (1978) Cryosurgery in the management of rectal tumours. *British Journal of Surgery*, **65**, 859–861
4. Cohen, J.K. and Onik, G. (1992) Cryosurgical ablation of the prostate: The Lazarus syndrome. Society of Cryobiology Annual Meeting 1992, Cryobiology A76 (in press)
5. Marcove, R.C. and Healey, J. (1992) Treatment of bone tumours by cryosurgery. Society of Cryobiology Annual Meeting 1992, Cryobiology A78 (in press)
6. Ravikumar, T.S., Kane, R., Cady, B., *et al.* (1987) Hepatic cryosurgery with intraoperative ultrasound monitoring for metastatic colon carcinoma. *Archives of Surgery*, **122**, 403–409
7. Onik, G., Rubinsky, B., Zemel, R. *et al.* (1991) Ultrasound-guided hepatic cryosurgery in the treatment of metastatic colon carcinoma. *Cancer*, **67**, 901–907
8. Zhou, X-D, Tang, Z-Y. Yu, Y-Q and Ma, Z-C. (1988) Clinical evaluation of cryosurgery in the treatment of primary liver cancer. Report of 60 cases. *Cancer*, **61**, 1889–1892
9. Bastid, C. (1991) L'alcoolisation percutanée guidée par échographie des tumeurs hépatiques. *Gastroenterol. Clin. Biol.*, **15**, T2–T3

